# 重组人血管内皮抑制素联合放射治疗对lewis肺癌小鼠的作用

**DOI:** 10.3779/j.issn.1009-3419.2010.04.22

**Published:** 2010-04-20

**Authors:** 渡 何, 伟 戈, 长虎 李, 振宇 赵, 细明 徐, 芳 杨

**Affiliations:** 1 430060 武汉，武汉大学人民医院肿瘤科 Department of Medical Oncology, Remmin Hospital of Wuhan University, Wuhan 430060, China; 2 430060 武汉，武汉大学基础医学院 College of Base Medicine, Wuhan University, Wuhan 430071, China

**Keywords:** 肺肿瘤, 放射治疗, 重组人血管内皮抑制素, Lung neoplasms, Radiotherapy, Rh-endostatin

## Abstract

**背景与目的:**

放疗敏感性与组织中氧含量关系密切，单用重组人血管内皮抑素（rh-endostatin，YH-16，恩度）可能通过血管“正常化”作用使组织中氧含量增加，这与放疗的敏感性密切相关。本研究将重组人血管内皮抑素与放射治疗联合对lewis肺癌小鼠的肿瘤生长及血管内皮生长因子（vascular endothelial growth factor, VEGF）表达的影响。

**方法:**

制作lewis肺癌细胞种植肿瘤的动物模型，小鼠随机分为四组：A组为空白对照组，B组为重组人血管内皮抑素组，C组为放疗组，D组为联合组。分别给予相应处理后绘制肿瘤生长曲线，计算抑瘤率，用免疫组化（SP法）测定VEGF的表达及微血管密度。

**结果:**

经过相应的处理后，各处理组较A组移植瘤的生长速度明显减慢。且各组瘤质量明显低于A组（*P* < 0.05），D组较其它三组降低得更加明显（*P* < 0.05），VEGF的表达及微血管密度与A组相比也有不同程度的降低（*P* < 0.05），其中D组下降最为明显。

**结论:**

重组人血管内皮抑素和放疗联用对lewis肺癌小鼠有明显肿瘤抑制作用，其机制可能是下调VEGF的表达、抑制新生血管的生成。

## Abstract (en)

**Background and objective:**

Radiation sensitivity is closely related to tissue oxygen, and rh-endostatin can induce the high level of oxygen content in tumor by " normalizing" tumor angiogenesis which is associated with radiotherapy sensitivity. The aim of this study is to observe the effect of combination of radiotherapy with rh-endostatin in the rats with lung cancer.

**Methods:**

Immediate lewis cancerous ascetic injection method was used to make rats tumors bearing model, then the rats was divided into four groups randomly: group A was treated with saline; group B was treated with rh-endostatin; group C was treated with irradiation and group D was treated with rh-endostatin and irradiation. After all rats were treated, inhibition rates and the tumor growth curve were calculated. Immunohistochemisty was adopted to check the expressions of vascular endothelial growth factor (VEGF) and microvessel density (MVD).

**Results:**

Compared with group A, the growth rates of the tumors in the other group were obviously slower, and the tumor weights were significantly different form group A (*P* < 0.05). Compared with the other groups, the tumor weights of group D were obviously reduced (*P* < 0.05). Compared with group A, VEGF and MVD of other three groups were reduced (*P* < 0.05), and group D were significantly cut down.

**Conclusion:**

Combination with radiotherapy and rh-endostatin could inhibit the lung cancer significantly in rats.The possible mechanisms are to decrease the expression of VEGF and inhibit the production of angiogenesis.

肺癌是国内外发病率与死亡率都非常高的恶性肿瘤，放射治疗是治疗肺癌的主要手段之一，但是单纯的放射治疗的效果尚不理想。因此，国内外一直在试图探寻一种新的、有效的联合治疗手段来提高放疗的疗效。本研究将已经在国内上市的抗血管生成药物——重组人血管内皮抑素（rh-endostatin，YH-16，恩度）与放疗联合，与传统的放疗方法和单独使用重组人血管内皮抑制素的方法进行疗效对比，观察对lewis肺癌小鼠生长及血管内皮生长因子（vascular endothelial growth factor, VEGF）表达的影响。

## 1 材料与方法

### 1.1 主要材料

#### 1.1.1 试剂

新生小牛血清购自杭州四季青公司，DMEM培养基及胰蛋白酶购自GIBCO公司，重组人血管内皮抑制素由先声药业公司提供，鼠VEGF抗体和鼠CD34 lgG购自武汉博士德生物公司。

#### 1.1.2 动物

lewis肺癌细胞，中国科学研究院药理研究所提供; 6周-8周、体重18 g-20 g的雌性C57 BL/6小鼠40只，购自武汉大学试验动物中心。

#### 1.1.3 仪器设备

山东新华Fcc-8000F型Co60治疗机，美国SHELDM2300型二氧化碳培养箱，北京海博川科技有限公司超净工作台，日本Olympus IMT-2倒置相差显微镜，HPLAS-1000高清晰彩色病理图文分析系统。

### 1.2 方法

#### 1.2.1 动物模型与相应处理

将生长良好的lewis肺癌细胞制成1×10^7^个/mL细胞悬液，0.2 mL/只接种于每只预先经脱毛和消毒的小鼠右后肢大腿腹侧。用游标卡尺测量肿瘤的最长直径和最短直径，并取其平均值，当肿瘤长至0.8 cm时将40只小鼠随机分为四组，每组10只。A组为空白对照组，每日尾静脉注射生理盐水0.1 mL; B组为重组人血管内皮抑素组，每日尾静脉注射浓度为10%的重组人血管内皮抑素0.1 mL，共15天; C组为放疗组; D组为联合组，每日尾静脉注射浓度为10%重组人血管内皮抑素0.1 mL（20 μg），共15天，在第6天-10天时给予放疗。

在放疗时，小鼠先用乙醚麻醉，待药物显效后将小鼠固定在自制的木板上，在右侧腹股沟肿瘤区给予Co^60^照射，剂量率为113 cGy/min，肿瘤区覆盖0.5 cm的组织等效材料，源皮距SSD为80 cm，剂量10 Gy/5次，5次/周。

#### 1.2.2 肿瘤体积及抑瘤率的测定

隔日一次用游标卡尺测量荷瘤小鼠最长径a（mm）和最短径b（mm），根据公式V=ab^2^/2计算肿瘤体积（V），并绘制肿瘤生长曲线。在处理结束后第10天处死小鼠，剥离瘤体，称重，计算抑瘤率。

抑瘤率=（对照组瘤质量-试验组瘤质量/对照组瘤质量）×100%。

#### 1.2.3 VEGF测定

肿瘤标本取下后立即用10%的中性甲醛固定4 h-8 h，脱水，石腊包埋后切片，应用常规免疫组织化学（SP法）检测VEGF的表达。结果判断：VEGF的阳性染色为胞质着色。光镜下可见细胞胞浆内出现棕黄色颗粒，棕黄色越多，染色越深，其表达也越强。采用HPLAS-1000彩色病理图文分析系统处理图片。

#### 1.2.4 微血管计数（microvessel density, MVD）

参照Veidner等报道的方法进行微血管计数。

#### 1.2.5 统计学处理

应用SPSS 13.0统计分析软件进行分析，计量资料的均数以Mean±SD表示; 多组均数比较方差齐性时采用单向方差分析，均数间两两比较采用*q*检验，方差不齐时则用非参数检验，以*P* < 0.05为有统计学差异。

## 2 结果

### 2.1 不同治疗组对lewis肺癌小鼠生长的影响及抑瘤率

在接种的第7天-9天时接种部位可及黄豆大小的硬块，肿瘤体积随着时间的增加逐渐变大，其中对照组肿瘤生长最为迅速，重组人血管内皮抑素组、放疗组和联合组三组的肿瘤生长都有不同程度的减慢（图 1）。荷瘤小鼠处死后，联合组瘤质量的平均值明显低于其它三组（*P* < 0.05），抑瘤率为63.34%±4.89%（表 1），高于重组人血管内皮抑素组和放疗组（*P* < 0.05），结果显示联合处理的抑瘤作用最强。

**1:**
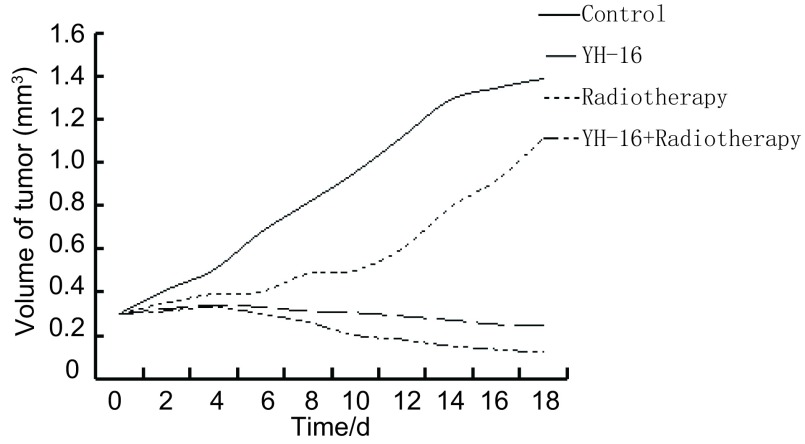
荷瘤小鼠治疗期间的肿瘤体积变化 The volume change in the rats with lung cancer during treatment

### 2.2 不同治疗组对VEGF的影响

免疫组化结果表明，肿瘤组织VEGF的阳性表达主要分布于胞浆，四组均可见VEGF的阳性表达，放疗组VEGF着色较深，呈强阳性表达（VEGF平均灰度值=146.67±2.29），重组人血管内皮抑素组和联合组VEGF着色均较对照组浅，其中联合组VEGF灰度值仅为42.13±3.59，与其它三组比较有统计学差异（*P* < 0.01）（图 2）。

**2:**
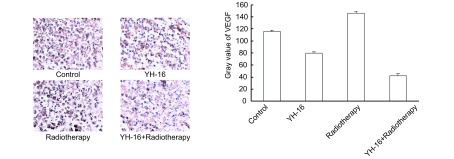
四个治疗组VEGF免疫染色（SP, ×200） Immuno-staining of VEGF in four groups (SP, ×200) VEGF: vascular endothelial growth factor.

### 2.3 不同治疗组对肿瘤微血管密度的作用

镜下观察，可见放疗组的血管增生明显（MVD=39.44±1.12）且可见血管基底膜不完整。重组人血管内皮抑素组（MVD=12.78±0.97）和联合组（MVD=6.22±0.36）血管生成减少，MVD值低于对照组（P < 0.05），且血管基底膜较完整。其中联合组新生血管生成极少（MVD=6.22±0.36），明显低于其它三组（*P* < 0.05）（图 3）。

**3:**
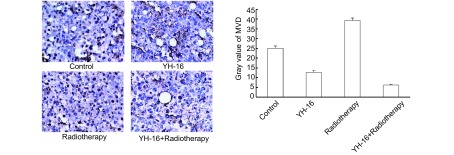
四个治疗组经过CD34染色的肿瘤微血管（SP, ×200） Tumor microvessel density in four group marked by CD34 (SP, ×200)

## 3 讨论

实体瘤因缺氧而导致新生血管的形成是肿瘤生长和转移的基础，同时也是肿瘤预后不良的重要因素^[1]^。近年来有学者提出的抗血管生成治疗有望成为抑制肿瘤生长又一突破口，并逐渐成为肿瘤研究领域所关注的新的热点^[2]^。目前内皮抑素（endostatin）是最强的血管生成抑制因子，在体内试验中，从大肠杆菌中得到的重组鼠内皮抑素蛋白，已经发现通过每天给药可以抑制异位移植鼠如lewis肺癌、T241骨纤维肉瘤或B16F10黑色素瘤的生长^[3]^。最近也有研究^[4]^报道经腺病毒转染的内皮抑素对lewis肺癌小鼠的生长和转移有明显的抑制作用。

目前的研究表明，在人体环境中，肿瘤可能存在数月或者数年，已经建立了一个可能对抗血管生成治疗并不敏感的血管系统，因此会影响临床治疗肿瘤疗效，因此学者们尝试将抗血管生成治疗和其它治疗联用以提高肿瘤治愈率。国内王金万、孙燕等^[6]^率先将重组人血管内皮抑素和NP方案联用治疗非小细胞肺癌，结果发现重组人血管内皮抑素+NP方案组较单用NP方案组有效率（response rate, RR）提高了16.1%，中位疾病进展时间（time to progression, TTP）也延长了2.9个月，表明重组人血管内皮抑素与NP方案具有协同作用，且不明显增加化疗的不良反应。本研究也表明重组人血管内皮抑素和放疗对抑制肿瘤生长和瘤质量有一定影响，但是联合组抑制作用更为显著，提示抗血管生成治疗和其它治疗（如放疗）联用可能是增加其疗效的有效途径。传统的治疗方法和抗血管生成治疗的联合可以通过靶向作用于肿瘤细胞和内皮细胞，从而提高肿瘤疗效。这种联合治疗策略的其中一个优势在于可以减低个体的药物剂量，但并不减弱抗肿瘤的疗效。已经有研究^[1]^表明血管生成治疗和化疗联用可以增加化疗药物在肿瘤组织中的药物浓度。关于抗血管生成治疗和放射治疗联用治疗肿瘤目前已经有一些相关的研究报道，Luo等^[7]^通过构建pXLG-mEndo质粒研究内源性小鼠内皮抑素联合放疗对肺癌模型的影响，试验表明，与其它组相比，pXLG-mEndo+放疗组提高了肿瘤组织内内皮抑素的水平，且小鼠的肿瘤生长得到了显著的抑制。Shibuya研究^[8]^也表明ZD6474（一种抗血管生成药物）可以降低肿瘤组织中VEGF及EGFR的表达水平，从而增加了非小细胞肺癌对放疗的敏感性。

对于局部晚期非小细胞肺癌，放疗为其主要治疗手段，但是这类患者经放疗后5年生存率仍不令人满意。2002年10月重组人血管内皮抑素正式进入Ⅱ期临床试验，同年Miren等^[9]^将人重组内皮抑素注入到51b-CC小鼠体内，结果发现肿瘤体积降低了40%。且2004年苏黎红等^[10]^研究重组人血管内皮抑素对荷瘤小鼠的影响，并确定了治疗小鼠的有效剂量及用药时间。这些试验数据为本研究的顺利进行提供了试验基础。

本研究中放疗组VEGF的表达（146.32±2.29）和MVD（39.44±1.12）水平与对照组相比显著增加，表明放疗可以提高肿瘤组织VEGF的表达水平，进而刺激肿瘤新生血管生成。Inoue研究^[11]^也发现直肠癌病人放疗后VEGF表达明显增加。其具体机制有待于进一步研究，可能与放疗引起乏氧细胞的比率增加有关，但是新生血管的增加并不一定相应地增加血流量和氧的供应，这是由于肿瘤的新生血管是异常的，新生血管内皮细胞与正常的血管相比有不同表型和功用，同时VEGF可以保护内皮细胞免受放射线的损伤，这些都是产生放射耐受的重要原因。Jain^[12]^提出抗血管治疗可以导致肿瘤血管在被破坏之前“正常化”。“正常化”的肿瘤血管与正常血管功能相似，使肿瘤氧水平高，乏氧得以改善，减少对放疗耐受的乏氧细胞的数量，提高了肿瘤细胞对放射的敏感性。本研究中联合组瘤质量（0.921±0.135）、MVD（6.22±0.36）和VEGF（42.13±3.59）的表达与其它三组相比均有明显下降，而且肿瘤血管的形态接近于正常的血管，表明重组人血管内皮抑素可能是通过使血管“正常化”，减少乏氧细胞的数量，从而使VEGF的表达下调，增加放疗敏感性。

综上所述，重组人血管内皮抑素联合放疗可以明显抑制lewis肺癌小鼠的肿瘤生长，通过间接下调VEGF的表达可能为其有效的途径之一。但是关于两者联合治疗的时序问题尚需进一步研究，本实验研究结果将为今后的关于抗血管生成治疗和放疗治疗联合运用提供实验依据，有助于进一步完善肿瘤治疗的临床解决方案。
